# Motivational, emotional, and cognitive profiles of dysregulated sexual behavior: a multilevel exploratory study

**DOI:** 10.1016/j.abrep.2026.100696

**Published:** 2026-04-12

**Authors:** Cécile Miele, Julien Cabé, Bruno Pereira, Valérie Moulin, Servane Barrault

**Affiliations:** a Laboratoire QualiPsy UR 1901, Université de Tours, 3 rue des Tanneurs, BP 4103 – 37041 Tours Cedex 1, France; b Service d’Addictologie et de pathologies duelles, Pôle de psychiatrie, CHU de Clermont-Ferrand, CMP B, 58 rue Montalembert, 63003 Clermont-Ferrand Cedex, France; c Université Clermont Auvergne, Clermont Auvergne INP, CHU Clermont-Ferrand, CNRS, Institut Pascal, F-63000 Clermont-Ferrand, France; d Direction de la Recherche Clinique et de l’Innovation, CHU de Clermont-Ferrand, 58 rue Montalembert, 63003 Clermont-Ferrand Cedex, France; e Laboratoire Inter-universitaire de Psychologie Personnalité, Cognition, Changement Social (LIP/PC2S), Université de Grenoble Alpes, BP 47 – 38040 Grenoble Cedex 9, France; f Centre de Soins d’Accompagnement et de Prévention en Addictologie (CSAPA 37), CHRU of Tours, Tours, France

## Abstract

**Background:**

Dysregulated sexual behavior (DSB) is clinically heterogeneous and may involve partially distinct motivational, emotional, and cognitive processes. This exploratory study examined whether descriptive profiles could be observed across these three domains in adults reporting loss of control over sexual behavior.

**Methods:**

In this cross-sectional exploratory study, 76 French adults reporting DSB were recruited through clinical and non-clinical channels. Participants completed self-report measures assessing sexual dysregulation, psychological distress, impulsivity, emotional reactivity, emotion regulation difficulties, alexithymia, cognitive distortions, and cognitive insight. Multiple correspondence analyses followed by hierarchical cluster analyses were conducted separately at the motivational/dynamic, emotional, and cognitive levels, and bootstrap-based stability analyses were used to assess the robustness of the clustering solutions.

**Results:**

Descriptive groupings were observed at each level, with three motivational profiles, four emotional profiles, and three cognitive profiles retained in this sample. Motivational profiles were suggestive of reward sensitivity, distress-related coping, and more habitual or cognitively maintained patterns. Emotional profiles reflected either greater emotion-processing limitations, heightened emotional reactivity, or more preserved regulation. Cognitive profiles differed mainly in the degree of cognitive distortions and cognitive insight. These profiles were only weakly associated with behavioral modalities such as paraphilic versus normophilic practices, cybersexual activity, and sexual behavior associated with psychoactive substance use. Bootstrap-based stability analyses supported the overall robustness of the retained solutions.

**Conclusions:**

A multilevel descriptive approach highlighted heterogeneous psychological configurations among French adults reporting DSB. Although these findings do not establish a validated typology, they suggest that distinguishing motivational, emotional, and cognitive domains may help generate clinically relevant hypotheses and inform future research on heterogeneity in dysregulated sexual behavior.

## Highlights

• Adults reporting dysregulated sexual behavior (DSB) showed distinct profiles across motivational, emotional, and cognitive domains.
• Three motivational profiles were described: stimulation-oriented, distress-responsive, and habit-maintained.
• Emotional and cognitive profiles varied across DSB, distinguishing alexithymia/reactivity patterns and levels of insight.
• Behavioral modalities (paraphilic, cybersexual, substance-linked) showed little association with psychological profiles.
• A multilevel descriptive approach may support process-oriented, individualized case formulation beyond behavior-centered classification.

## 1 Background

### 1.1 Conceptual background

Loss of control over sexual thoughts, urges, or behaviors has been described as a clinically heterogeneous phenomenon. Individuals reporting such difficulties may present with diverse behavioral patterns and partially distinct psychological processes.

In this context, although Compulsive Sexual Behavior Disorder (CSBD) is the current diagnostic term adopted in ICD-11, the present study uses dysregulated sexual behavior (DSB) as a broader functional term of analysis, in order to examine clinically significant loss-of-control sexual behaviors without presupposing a single underlying mechanism or a strict fit within one nosographic category. This choice also reflects the broader lack of consensus regarding the most appropriate terminology and conceptual framing for this phenomenon (Griffin et al., 2021, Reid, 2016).

Under various labels, including compulsive sexual behavior, hypersexuality, sexual addiction, and problematic sexual behavior, this clinical field has long been described as heterogeneous in both its manifestations and its associated psychological correlates (Griffin et al., 2021, Reid, 2016). Some explanatory perspectives emphasize compulsive features and regard sexual behavior primarily as a maladaptive strategy for regulating distress or aversive affective states (Grubbs et al., 2020, Lew-Starowicz et al., 2020). Others highlight impulsive mechanisms, including heightened reward sensitivity, sensation seeking, and deficient inhibitory control (Bocci Benucci et al., 2024, Bőthe et al., 2019). Still others, drawing on addiction models, stress the role of reinforcement, craving, conditioning, and habit formation in the persistence of problematic sexual behavior (Grubbs et al., 2020, Kingston, 2015). Rather than viewing these perspectives as mutually exclusive, it may be more useful to consider that similar behavioral outcomes can arise from partially distinct functional configurations (Ballester-Arnal et al., 2020, Grubbs et al., 2020).

Earlier work has also suggested that problematic sexual behavior may be better understood through multiple functional dimensions than through a single unitary mechanism. In particular, clinically oriented models have highlighted the relevance of dimensions such as impaired control, coping-related use of sex, and adverse consequences (Reid et al., 2011). Such perspectives remain informative because they suggest that heterogeneity may not simply concern the content of sexual behavior, but also the processes through which it is regulated, maintained, and experienced.

### 1.2 Behavioral heterogeneity and clinical scope

A central difficulty in this field lies in the diversity of behaviors that may be experienced as dysregulated. These may include compulsive masturbation, excessive pornography use, cybersexual activity, compulsive partner seeking, repetitive seduction, and, in some cases, paraphilic practices (Castro-Calvo et al., 2020, Kafka, 2010). Such diversity complicates efforts to construct explanatory models based solely on behavioral description. The same overt sexual behavior may serve different functions across individuals, while similar functional processes may be expressed through different sexual behaviors. This suggests that classifications centered primarily on behavioral content may not be sufficient to capture the psychological heterogeneity of DSB (Grubbs et al., 2020).

Some individuals reporting DSB may also report paraphilic practices, which may coexist with other forms of dysregulated sexual behavior within the same clinical presentation (Briken et al., 2024, Culos et al., 2024, Kafka and Hennen, 2003). Although paraphilic interests and DSB are conceptually distinct (Briken et al., 2024, Krueger et al., 2017, Reed et al., 2016), paraphilic versus normophilic practices were treated here as descriptive behavioral modalities rather than as markers of separate nosographic entities (Brown et al., 2023, Castellini et al., 2018, Reed et al., 2016). This choice reflects the study’s focus on functional heterogeneity rather than on categorizing sexual contents.

### 1.3 Multilevel rationale and study aims

In response to such heterogeneity, a multilevel descriptive approach may offer a useful exploratory strategy. Clinically informed typologies have attempted to distinguish recurring presentations of problematic sexuality, but these often remain centered on observed forms of behavior or broad clinical impressions (Cantor et al., 2013). Recent person-centered research has likewise suggested that pornography-use-related problems may involve distinguishable profiles shaped by multiple clinically relevant dimensions, reinforcing the broader view that apparently similar sexual difficulties may not be reducible to a single process (Bőthe et al., 2025). By contrast, work in behavioral addictions, particularly gambling disorder, has suggested that similar problematic behaviors may be associated with partially distinct pathways (Blaszczynski & Nower, 2002). This perspective is relevant here not because a pathways model can be directly transposed to DSB, but because it provides a heuristic way of thinking about heterogeneity in terms of multiple coexisting configurations rather than a single explanatory mechanism (Blaszczynski and Nower, 2002, Nower et al., 2022).

From this perspective, motivational or dynamic processes constitute a first relevant level of analysis. Dysregulated behaviors may be associated with different functional dynamics, including reward seeking, relief seeking, urgency, and habit-like maintenance (Golder et al., 2023, Lew-Starowicz et al., 2020). Applied to DSB, this suggests that loss of control over sexual behavior may not always reflect the same motivational organization. For some individuals, the behavior may be more strongly associated with novelty, excitement, and immediate reward; for others, with coping with distress or aversive affect; and for others still, with increasingly automated or cognitively maintained patterns. These possibilities do not amount to distinct theories to be tested against one another, but rather indicate that motivational heterogeneity may be clinically and theoretically meaningful.

A second relevant level concerns emotional functioning. Emotional dysregulation has repeatedly been implicated in both addictive behaviors and DSB (Grant Weinandy et al., 2023, Lew-Starowicz et al., 2020). However, emotional dysregulation is not a unitary construct. Difficulties may arise at different stages of emotional functioning, including emotional awareness, identification, clarity, acceptance, modulation, and behavioral control under emotional load (Gori and Topino, 2024, Preece et al., 2023). Alexithymia may further complicate this picture by limiting the capacity to identify and symbolize emotional states, thereby increasing reliance on action-based forms of regulation (Preece et al., 2023, Reid et al., 2008). Exploring emotional heterogeneity may therefore help distinguish between different forms of difficulty within DSB rather than treating emotional dysregulation as a single dimension.

A third relevant level concerns cognitive regulation. Cognitive theories of problematic and addictive behaviors have emphasized the role of maladaptive beliefs and cognitive distortions in the initiation, justification, and maintenance of dysregulated behaviors (Beck et al., 1993, Hautekeete et al., 1999). In DSB, such cognitions may include anticipatory beliefs about pleasure or relief, permissive justifications, minimization of negative consequences, or other distorted appraisals that facilitate enactment and reduce self-questioning (Efrati et al., 2021). Cognitive insight may also be relevant, as individuals may differ in their capacity to critically examine their own interpretations and beliefs (Beck et al., 2004, Marchetti, 2023). Considering this level separately may therefore help document additional forms of heterogeneity that are not reducible to motivational or emotional factors alone.

Taken together, these considerations support the relevance of a multilevel descriptive perspective on DSB (Ballester-Arnal et al., 2020, Grubbs et al., 2020). Rather than assuming a single explanatory mechanism, such an approach seeks to explore whether different configurations can be observed across complementary domains of functioning. In this sense, the aim is not to validate a formal typology or resolve nosographic debates, but to examine whether a descriptive mapping of heterogeneity may be clinically and theoretically informative.

The present exploratory study therefore examined whether distinct descriptive profiles could be observed at three complementary levels of functioning in adults reporting DSB: (i) motivational/dynamic processes, (ii) emotional regulation, and (iii) cognitive regulation. A secondary aim was to explore whether these descriptive profiles were associated with behavioral modalities such as paraphilic versus normophilic practices, cybersexual activity, and sexual behavior associated with psychoactive substance use. Rather than testing a formal typological model, the study was intended to generate clinically and theoretically relevant hypotheses about heterogeneity within DSB.

## 2 Method

### 2.1 Study design and participants

This was a cross-sectional exploratory descriptive study involving 76 adults reporting repetitive sexual behaviors experienced as difficult to control. Participants were recruited through both clinical and non-clinical channels in France. Clinical recruitment took place in addiction medicine and sexual health departments of several French university hospitals, where eligible patients were informed about the study by their caregivers. Non-clinical recruitment relied on dissemination of an online questionnaire link through social networks and educational activities conducted by the research team.

Inclusion criteria were age over 18 years and self-reported DSB, whether involving normophilic or paraphilic practices. This broad inclusion criterion was chosen to capture clinical heterogeneity within a function-oriented exploratory framework rather than to approximate a specific diagnostic category. Individuals who had committed contact sexual violence were excluded. All participants provided free and informed consent, and the study protocol was approved by a regional university ethics committee (No. CER-TP 2022-01-05).

### 2.2 Measures

All variables were assessed using French self-report questionnaires compiled into a single online booklet. Sociodemographic data and descriptive information regarding sexual behaviors were collected using an ad hoc questionnaire, including type of practices, frequency, online activity, associated psychoactive substance use, and presence of paraphilic behaviors.

DSB was screened using the Sexual Addiction Screening Test (SAST), a historically influential and widely used instrument in the field of problematic sexual behaviors (Hegbe et al., 2020). In the present study, the SAST was used as a pragmatic symptom-screening tool rather than as an endorsement of a specific addiction-based conceptualization. Sexual craving was assessed phenomenologically as the subjective experience of recurrent urges and felt pull toward sexual behavior. In the present study, craving was considered a clinically relevant experiential dimension of loss of control rather than a construct specific to an addiction model.

Variables selected to explore the motivational/dynamic level included indicators related to sensation seeking, urgency, and psychological distress. Anxiety and depressive symptoms were measured using the Hospital Anxiety and Depression Scale (HADS/HAD) (Lépine et al., 1985). Impulsivity was assessed using the short version of the UPPS-P scale (Billieux et al., 2012), measuring negative urgency, positive urgency, sensation seeking, lack of perseverance, and lack of premeditation.

Emotional regulation was explored through three complementary instruments: the Emotion Reactivity Scale (ERS) (Lannoy et al., 2014), the Difficulties in Emotion Regulation Scale (DERS) (Côté et al., 2013), and the Toronto Alexithymia Scale (TAS-20) (Loas et al., 1995). Together, these measures were used to document emotional sensitivity, modulation capacities, and difficulties in emotional identification and expression.

Cognitive regulation was examined using an adapted version of the Anticipatory–Relief–Permissive cognitive distortions scale (ASP; Anticipatoire–Soulageante–Permissive in French) (Hautekeete et al., 1999), originally developed for alcohol use disorders and grounded in Beck’s cognitive model of addiction. In the present study, the scale was adapted to assess cognitive distortions related to sexual behavior. Cognitive insight was measured using the Beck Cognitive Insight Scale (BCIS) (Favrod et al., 2008), which distinguishes self-reflectiveness from self-certainty.

### 2.3 Statistical analyses

Given the exploratory nature of the study and the use of multivariate techniques (multiple correspondence analysis followed by clustering), no formal a priori sample size or power calculation was performed, as these methods are not centered on a single hypothesis test with a predefined effect size. Instead, emphasis was placed on evaluating the stability and internal consistency of the clustering solutions. The available sample size, relative to the number of variables included in each analysis, may be considered modest, and no formal sample size recommendations exist for this type of exploratory multivariate approach. To mitigate this limitation, we explicitly evaluated the stability and reproducibility of the clustering solutions using bootstrap-based methods. This sample size may nevertheless limit the generalizability of the findings.

Descriptive statistics were computed for all variables. Categorical variables were expressed as frequencies and percentages, and continuous variables as means and standard deviations or medians and interquartile ranges depending on distribution. Normality was assessed using the Shapiro–Wilk test.

Univariate analyses examined associations between behavioral modalities (paraphilia, cybersexual activity, and associated substance use) and psychological variables using chi-square or Fisher’s exact tests as appropriate.

Rather than integrating all variables into a single multivariate model, we deliberately adopted a multilevel analytical strategy, conducting separate typological analyses at the motivational, emotional, and cognitive levels. This choice was theoretically and clinically motivated, as it allowed preservation of process specificity and avoided collapsing distinct mechanisms into a single latent construct. From a clinical perspective, this approach was intended to maximize the therapeutic relevance of the typology by enabling identification of differentiated intervention targets across levels of functioning.

To identify typological profiles, multiple correspondence analyses (MCA) were conducted separately at the dynamic, emotional, and cognitive levels. The number of retained dimensions was determined based on eigenvalues, scree plot inspection, and the proportion of explained variance. Hierarchical cluster analyses were subsequently applied to the MCA dimensions to identify groups of individuals sharing similar patterns, using Ward’s method with Euclidean distance. The optimal number of clusters was determined using a combination of criteria, including inspection of dendrograms, evaluation of inertia gain (elbow criterion), and comparison of alternative cluster solutions. The final solution was selected based on the coherence and interpretability of the clusters, as well as their clinical relevance. Cluster solutions with very small group sizes were not retained. Furthermore, to assess the robustness of the clustering solutions, we performed a bootstrap-based stability analysis using Jaccard similarity coefficients (1000 resamples). In addition, sensitivity analyses were conducted (e.g., variations in clustering initialization and/or retained dimensions) to examine the consistency of the identified clustering structures under alternative analytical specifications. This approach allows evaluation of the reproducibility of clusters under sampling variability, which is particularly relevant in exploratory typological analyses where conventional statistical power is not directly applicable.

Statistical analyses were performed using Stata software (version 15, StataCorp, College Station, Texas, US) and R 3.3.3 (http://cran.r-project.org/). All the tests were two-sided, with a Type I error set at 0.05.

## 3 Results

### 3.1 Descriptive findings

#### 3.1.1 Demographic and sexual practices characteristics (Table 1)

The sample consisted predominantly of men (81.6%), with a mean age of 38.9 years (SD = 13.4). Reported sexual behaviors were heterogeneous and included compulsive partner seeking, masturbation, pornography consumption, cybersexual activities, and paraphilic practices. More than one third of participants reported paraphilic behaviors (35.5%), and nearly one third reported sexual practices associated with psychoactive substance use (28.9%). Regardless of frequency or modality, all participants reported experiencing their sexual behavior as difficult to control.

**Table 1:** Main descriptive characteristics of the sample.

Variables	n (%)	Mean ± sd
**Gender**		
Female	12 (15.8)	_
Male	62 (81.6)	_
Other	2 (2.6)	_
**Age**	_	38.9 ± 13.4
**Marital situation**		
Single /separated /divorced	39 (51.3)	
Married / in couple	37 (48.7)	
**Professional situation**		_
Active, student	59 (77.6)	
Unemployed, retired	17 (22.4)	
**Sexual behavior**		
Paraphilic	27 (35.5)	_
Cyber exclu	11 (14.5)	_
Cyber mixt	18 (23.7)	_
Psychoactive substance (PaS)	22 (28.9)	_
**Frequency**		_
<1/week	23 (30.3)	
1/week	7 (9.2)	
several/week	27 (35.6)	
several/day	19 (25)	

*Note.* PaS = psychoactive substance-associated sexual behavior; Cyber exclu = exclusive cybersexual behavior; Cyber mixt = mixed cybersexual and non-cybersexual behavior; frequency refers to reported frequency of the dysregulated sexual behavior.

Approximately two thirds of participants obtained a positive score on the Sexual Addiction Screening Test (SAST; 69.7%), whereas a larger proportion of the sample (80.3%) reported sexual craving. Neither indicator differed significantly according to behavioral modalities (Table 2).

**Table 2:** Main results of the univariate analysis for diagnostic variables.

Variables	TOTAL (n (%))	Cyber				Paraphilia			PaS associated		
		None	Mixt	Exclusive	P-value	No	Yes	p-value	No	Yes	p-value
**TOTAL**	**_**	**37 (48.7)**	**28 (36.8)**	**11 (14.5)**	**_**	**49 (64.5)**	**27 (35.5)**	**_**	**54 (71.1)**	**22 (28.9)**	**_**
SAST	**53 (69.7)**	25 (67.6)	21 (75)	7 (63.6)	0.725	35 (71.4)	18 (66.7)	0.665	40 (74.1)	13 (59.1)	0.197
Craving	**61 (80.3)**	30 (81.1)	20 (71.4)	11 (100)	0.129	40 (81.4)	21 (77.8)	0.686	43 (79.6)	18 (81.8)	0.828

*Note.* SAST = Sexual Addiction Screening Test; Craving = self-reported sexual craving; PaS = psychoactive substance-associated sexual behavior; Cyber exclus. = exclusive cybersexual behavior; Cyber mixt = mixed cybersexual and non-cybersexual behavior.

#### 3.1.2 Dynamic emotional and cognitive variables (Table 3)

At the motivational/dynamic level, high rates of anxiety and moderate rates of depression were observed (69.7% and 46.1%, respectively). Impulsivity scores were elevated across the UPPS-P dimensions, with particularly high scores on sensation seeking (10.8 ± 3.0) and positive urgency (11.4 ± 2.8). At the emotional level, moderate emotion regulation difficulties were observed across DERS dimensions, together with elevated rates of alexithymia. At the cognitive level, cognitive distortions were present but moderate overall, and cognitive insight scores were close to the sample average.

**Table 3:** Main descriptive results for psychological scales and subscales.

Variables (rank)	None | Doubt |Diagnostic n (%)	mean ± sd	median [Q1; Q3]	Min–max
HAD – Anxiety	23 (30,3) | 20 (26,3) | 33 (43,4)	_	_	_
HAD – Depression	41 (54) | 17 (22,4) | 18 (23,7)	_	_	_
UPPS-P – Nega Urg (4 > 16)	_	10.3 ± 3.3	10 [7; 13]	4–16
UPPS-P – Persev (4 > 16)	_	8 ± 2.7	8 [6; 10]	4–16
UPPS-P – Prem (4 > 16)	_	8 ± 2.7	8 [6; 9]	4–16
UPPS-P – Sensa (4 > 16)	_	10.8 ± 3	11 [9; 13]	5–16
UPPS-P – Posi Urg (4 > 16)	_	11.4 ± 2.8	12 [9; 13]	4–16
TAS – Descrip (5 > 25)	_	15.6 ± 5.1	15.5 [10.5; 20]	5–25
TAS – Identif (7 > 35)	_	19.4 ± 6.6	20 [14; 24]	8–35
TAS – Ext (8 > 40)	_	18.7 ± 4.6	18.5 [16; 21]	8–31
TAS − Total	33 (43.4) | 16 (21.1) | 27 (35.5)	_	_	_
ERS (0 > 84)	_	43 ± 17	43.5 [29.5; 53]	12–79
DERS – Awareness (6 > 30)	_	16.2 ± 4.5	17 [13; 20]	6–29
DERS – Clarity (5 > 25)	_	12.5 ± 5.2	12 [8; 16]	5–25
DERS – Control (6 > 30)	_	15.8 ± 6.2	15 [11.5; 20]	6–30
DERS – Engag (5 > 25)	_	16.4 ± 6.3	16 [12.5; 20]	6–25
DERS – Limit (8 > 40)	_	23.1 ± 8.7	22 [16.5; 30.5]	0–40
DERS – Acceptance (6 > 30)	_	16.4 ± 6.3	15.5 [12; 22]	6–29
ASP-sex – Anticip (0 > 40)	_	14.8 ± 8.2	14 [8; 18.5]	0–33
ASP-sex – Relief (0 > 40)	_	20.6 ± 8.9	22 [13; 27]	1–38
ASP-sex – Permissive (0 > 40)	_	14.4 ± 6.8	15 [9; 19.5]	0–28
BCIS – Self-reflex (0 > 27)	_	12.2 ± 4.7	12 [9; 15.5]	1–22
BCIS – Self-certain (0 > 18)	_	6.7 ± 3.3	7 [4; 9]	0–14
BCIS – Total (−18 > 27)	_	5.5 ± 5.4	6 [2.5; 8.5]	−7–17

*Note.* HAD = Hospital Anxiety and Depression Scale; UPPS-P = UPPS-P Impulsive Behavior Scale (Urgency, Premeditation, Perseverance, Sensation Seeking, Positive Urgency); Nega Urg = negative urgency; Persev = lack of perseverance; Prem = lack of premeditation; Sensa = sensation seeking; Posi Urg = positive urgency; TAS = Toronto Alexithymia Scale; Descrip = difficulty describing feelings; Identif = difficulty identifying feelings; Ext = externally oriented thinking; ERS = Emotion Reactivity Scale; DERS = Difficulties in Emotion Regulation Scale; Awareness = lack of emotional awareness; Clarity = lack of emotional clarity; Control = impulse control difficulties; Engag = difficulties engaging in goal-directed behavior; Limit = limited access to emotion regulation strategies; ASP-sex = adapted Anticipatory–Relief–Permissive cognitive distortions scale for sexual behavior; Anticip = anticipatory cognitions; Relief = relief-oriented cognitions; Permissive = permissive cognitions; BCIS = Beck Cognitive Insight Scale; Self-reflex = self-reflectiveness; Self-certain = self-certainty.

### 3.2 Typological analyses

#### 3.2.1 Dynamic profiles

Overall, the psychological variables showed little variation according to normophilic versus paraphilic practices, cybersexual activity, or sexual behavior associated with psychoactive substance use. The only significant association was that exclusively cybersexual participants showed fewer relief-oriented cognitive distortions (p < 0.05) (Table 4).

**Table 4:** Summary of the main results of the univariate analysis of dynamic-level variables.

Variables (rank)	TOTAL	Cyber			Paraphilia		PaS associated	
		None	Mixt	Exclusive	No	Yes	No	Yes
		37 (48.7)	28 (36.8)	11 (14.5)	49 (64.5)	27 (35.5)	54 (71.1)	22 (28.9)
**HAD depression**	no | doubt | diag (%)	59.5 | 16.2 | 24.3	39.3 | 35.7 | 25	72.7 | 9.1 | 18.2	57.1 | 20.4 | 22.5	48.2 | 25.9 | 25.9	57.4 | 20.4 | 22.2	45.5 | 27.3 | 27.3
**HAD anxiety**	no | doubt | diag (%)	32.4 | 21.6 | 46	25 | 35.7 | 39.3	36.4 | 18.2 | 45.5	26.5 | 28.6 | 44.9	37 | 22.2 | 40.7	33.3 | 24.1 | 42.6	22.7 | 31.8 | 45.5
**UPPS-P negative urg (4 > 16)**	mean ± sd	10.4 ± 3.2	10.5 ± 3.4	9.5 ± 3.5	9.8 ± 3.2	11.2 ± 3	10.3 ± 3.2	10.3 ± 3.5
	median [Q1; Q3]	10 [8; 13]	10 [7.5; 13]	8 [7; 12]	10 [7; 12]	11 [8; 14]	10 [8; 13]	11 [7; 13]
**UPPS-P perseverance (4 > 16)**	mean ± sd	7.3 ± 2.8	8.9 ± 2.6	7.8 ± 2.4	8.1 ± 2.9	7.8 ± 2.5	7.8 ± 2.5	8.3 ± 3.2
	median [Q1; Q3]	7 [5; 8]	8 [7; 10.5]	7 [6; 9]	8 [6; 10]	7 [6; 10]	8 [6; 9]	8 [6; 10]
**UPPS-P premeditation (4 > 16)**	mean ± sd	8.2 ± 3.1	7.8 ± 2.1	7 ± 2.3	7.7 ± 2.7	8.1 ± 2.6	7.8 ± 2.6	8 ± 3
	median [Q1; Q3]	8 [6; 10]	8 [6; 9]	7 [4; 8]	8 [6; 9]	8 [6; 10]	8 [6; 9]	8 [5; 9]
**UPPS-P sensation (4 > 16)**	mean ± sd	11.1 ± 3	11 ± 2.8	9.5 ± 3.7	10.8 ± 2.9	10.9 ± 3.4	10.5 ± 3	10.6 ± 3.1
	median [Q1; Q3]	11 [9; 13]	11 [9; 13.5]	9 [7; 13]	10 [9; 13]	11 [8; 14]	12 [9; 14]	11 [8; 13]
**UPPS-P positive urg (4 > 16)**	mean ± sd	11.2 ± 2.7	12.1 ± 2.8	10.8 ± 3.2	11.2 ± 2.7	11.8 ± 2.9	11.3 ± 3	11.9 ± 2.1
	median [Q1; Q3]	11 [9; 13]	13 [9.5; 14.5]	11 [9; 13]	12 [9; 13]	13 [10; 14]	11 [9; 14]	12 [11; 13]
**ASP-sex anticipatory (0 > 40)**	mean ± sd	16.6 ± 8.8	13.5 ± 7.1	12 ± 7.8	15.6 ± 8	13.3 ± 8.4	15.6 ± 8.6	12.8 ± 6.8
	median [Q1; Q3]	16 [10; 24]	13 [7.5; 17]	13 [6; 16]	15 [10; 20]	13 [6; 17]	14.5 [8; 23]	12 [8; 17]
**ASP-sex relief (0 > 40)**	mean ± sd	**22.8 ± 8.5** *	**19.6 ± 9** *	**15.5 ± 8.5** *	22.3 ± 8.1	17.4 ± 9.6	20.4 ± 9.6	21 ± 7.1
	median [Q1; Q3]	**26 [13; 28]** *	**19 [14.5; 19]** *	**16 [8; 24]** *	24 [15; 28]	17 [8–26]	21 [13; 28]	23 [15; 26]

*Note.* HAD = Hospital Anxiety and Depression Scale; UPPS-P = UPPS-P Impulsive Behavior Scale (Urgency, Premeditation, Perseverance, Sensation Seeking, Positive Urgency); negative urg = negative urgency; perseverance = lack of perseverance; premeditation = lack of premeditation; sensation = sensation seeking; positive urg = positive urgency; ASP-sex = adapted Anticipatory–Relief–Permissive cognitive distortions scale for sexual behavior; anticipatory = anticipatory cognitions; relief = relief-oriented cognitions; PaS = psychoactive substance-associated sexual behavior; Cyber none = no cybersexual behavior; Cyber mixt = mixed cybersexual and non-cybersexual behavior; Cyber exclusive = exclusive cybersexual behavior. *p < 0.05; **p < 0.01.

**Fig. 1:**
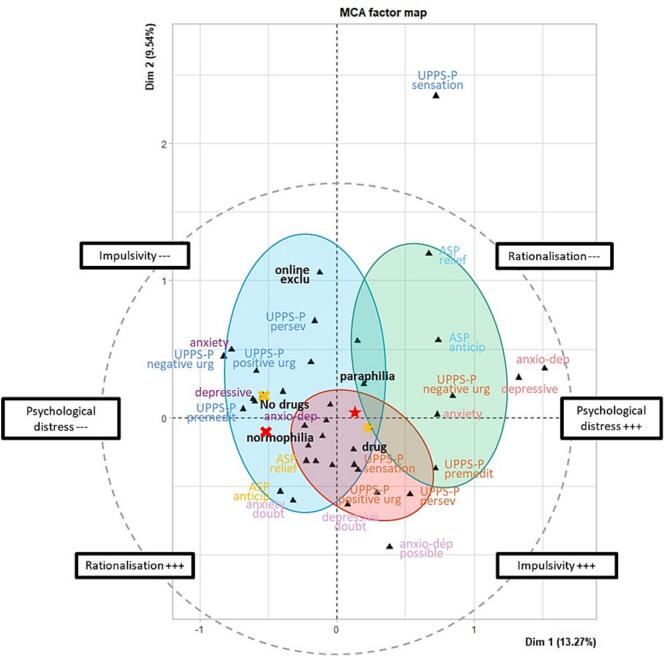
Multiple correspondence analysis factor map of the dynamic-level profiles. *Note.* MCA = Multiple Correspondence Analysis; UPPS-P = UPPS-P Impulsive Behavior Scale (Urgency, Premeditation, Perseverance, Sensation Seeking, Positive Urgency); ASP = Anticipatory–Relief–Permissive cognitive distortions scale (Anticipatoire–Soulageante–Permissive in French); anxiety/depressive/anxio-dep = anxiety, depressive, and mixed anxious-depressive symptoms assessed with the Hospital Anxiety and Depression Scale; no drugs/drug = sexual behavior not associated vs. associated with psychoactive substance use; normophilia/paraphilia = normophilic vs. paraphilic practices.

At the dynamic level, a three-group descriptive solution was retained (Fig. 1; Supplementary Information 1). For readability, heuristic labels were assigned to the profiles on the basis of their dominant score configurations.

The first profile, termed *stimulation-oriented* (n = 13; 17.1%), was distinguished by high sensation seeking (69.2% in the highest quartile) and high positive urgency (61.5% in the highest quartile). Participants in this group were also largely SAST-positive (92.3%) and reported craving in the large majority of cases (92.3%). Although this profile also included elevated distress indicators, its relative position on reward-related impulsivity variables supports a functional interpretation centered on novelty, excitement, and immediate reward.

The second profile, termed *distress-responsive* (n = 19; 25.0%), showed the highest levels of psychological distress, with diagnostic levels of depression in 84.2%, diagnostic anxiety in 94.7%, and combined anxiety–depression in 84.2% of participants. This group also showed the highest proportion of participants in the upper quartile for negative urgency (52.6%) and lack of premeditation (68.4%), together with high levels of craving (94.7%) and positive SAST status (89.5%). This configuration is consistent with a stronger association between sexual behavior and distress-related coping processes.

The third profile, termed *habit-maintained* (n = 44; 57.9%), was characterized by relatively preserved psychological functioning, with no combined anxiety–depression in all participants and no depressive symptoms in the large majority (86.4%). This group also showed the lowest proportion of positive SAST scores (54.6%) and the highest proportion of participants without craving (29.6%). Relative to the other profiles, it showed more moderate-to-high anticipatory and relief-oriented cognitions, suggesting a configuration in which sexual behavior may be maintained less by acute distress than by more established cognitive and behavioral patterns.

### 3.3 Sexual practices were not specifically associated with this profile

#### 3.3.1 Emotional profiles

Emotional variables did not differ significantly according to practice type or substance use, with one exception: lack of emotional acceptance was lower among participants reporting paraphilic practices than among those reporting normophilic practices (p < 0.01) (Table 5).

**Table 5:** Summary of the Main Results of the Univariate Analysis of Emotional-Level Variables.

Variables (rank)	TOTAL	Cyber			Paraphilia		PaS associated	
		None	Mixt	Exclusive	No	Yes	No	Yes
		37 (48.7)	28 (36.8)	11 (14.5)	49 (64.5)	27 (35.5)	54 (71.1)	22 (28.9)
**DERS clarity(5 > 25)**	mean ± sd	13.2 ± 5.9	11.7 ± 4	12.2 ± 5.4	12.6 ± 5.2	12.4 ± 5.3	12.7 ± 5.3	12.1 ± 5
	median [Q1; Q3]	14 [8; 17]	12 [8; 15]	11 [9; 14]	13 [8; 16]	11 [8; 16]	12.5 [8; 16]	11.5 [7; 16]
**DERS awareness(6 > 30)**	mean ± sd	16.2 ± 4.9	15.6 ± 4.1	17.9 ± 3.9	15.9 ± 4.4	16.8 ± 4.7	16.4 ± 4.6	15.7 ± 4.2
	median [Q1; Q3]	17 [13; 20]	15.5 [13; 19]	20 [13; 21]	16 [13; 20]	17 [13–20]	17 [13; 20]	16 [13; 19]
**DERS acceptance(6 > 30)**	mean ± sd	17.2 ± 6	15.8 ± 5.9	15.1 ± 8.4	16.9 ± 5.4**	15.5 ± 7.7**	15.9 ± 6.8	17.8 ± 4.9
	median [Q1; Q3]	18 [13; 22]	14.5 [11; 21.5]	13 [7; 25]	**16 [13; 21]**	**13 [9; 23]**	14.5 [10; 21]	18 [13; 22]
**DERS control(6 > 30)**	mean ± sd	16.5 ± 6.6	15.9 ± 5.7	13.2 ± 5.8	15.9 ± 5.7	15.6 ± 7	15.6 ± 6.3	16.1 ± 6.2
	median [Q1; Q3]	17 [12; 20]	16 [12.5; 19.5]	13 [8; 16]	16 [12; 20]	15 [9; 22]	17 [10; 20]	14.5 [12; 20]
**DERS engagement(5 > 25)**	mean ± sd	16.4 ± 5.4	16.4 ± 4.7	16.3 ± 4.8	16.6 ± 4.8	16.7 ± 5.1	16.3 ± 5	16.7 ± 5
	median [Q1; Q3]	16 [12; 20]	15.5 [13; 20.5]	18 [12; 21]	15 [13; 20]	16 [12; 21]	16 [13; 21]	16.5 [12; 20]
**DERS limited access(6 > 30)**	mean ± sd	23.9 ± 8.9	21.6 ± 7.7	24.5 ± 10.6	23.9 ± 8.8	27 ± 8.6	23.2 ± 8.7	23.1 ± 9
	median [Q1; Q3]	26 [19; 31]	20 [17; 29]	22 [14; 37]	25 [17; 31]	21 [14; 29]	22 [16; 40]	21 [19; 32]
**ERS(0 > 84)**	mean ± sd	43.8 ± 17.4	44 ± 15.7	38.1 ± 19.5	43.2 ± 16.5	42.7 ± 18.1	43.8 ± 17.6	41.1 ± 15.5
	median [Q1; Q3]	41 [29; 53]	44.5 [31; 52.5]	39 [15; 52]	40 [30; 53]	45 [28; 53]	44.5 [30; 58]	39 [29; 48]
**TAS identification(7 > 35)**	mean ± sd	19.9 ± 7.5	18.9 ± 6.1	19.2 ± 4.7	19.2 ± 7	19.8 ± 6	19.5 ± 6.8	19.3 ± 6.2
	median [Q1; Q3]	20 [14; 25]	20 [15; 23]	19 [15; 24]	20 [14; 24]	19 [15; 24]	20 [14; 24]	19.5 [14; 24]
**TAS description(5 > 25)**	mean ± sd	16 ± 5.7	14.9 ± 5.8	16.3 ± 5.2	15.3 ± 5.4	16.3 ± 6.1	15.9 ± 5.8	15 ± 5.2
	median [Q1; Q3]	18 [11; 21]	15.5 [10; 19.5]	15 [13; 20]	15 [11; 20]	18 [10; 22]	15.5 [11; 20]	15 [10; 19]
**TAS extern(8 > 40)**	mean ± sd	19 ± 4.7	18.3 ± 4.5	18.5 ± 5.2	18.3 ± 4.7	19.4 ± 4.5	19 ± 4.7	17.8 ± 4.5
	median [Q1; Q3]	20 [16; 22]	18.5 [16; 21]	16 [15; 25]	18 [16; 21]	19 [16; 21]	19 [16; 21]	18 [16; 21]

*Note.* DERS = Difficulties in Emotion Regulation Scale; clarity = lack of emotional clarity; awareness = lack of emotional awareness; acceptance = nonacceptance of emotional responses; control = impulse control difficulties; engagement = difficulties engaging in goal-directed behavior; limited access = limited access to emotion regulation strategies; ERS = Emotion Reactivity Scale; TAS = Toronto Alexithymia Scale; identification = difficulty identifying feelings; description = difficulty describing feelings; extern = externally oriented thinking; PaS = psychoactive substance-associated sexual behavior; Cyber none = no cybersexual behavior; Cyber mixt = mixed cybersexual and non-cybersexual behavior; Cyber exclusive = exclusive cybersexual behavior. *p < 0.05; **p < 0.01.

**Fig. 2:**
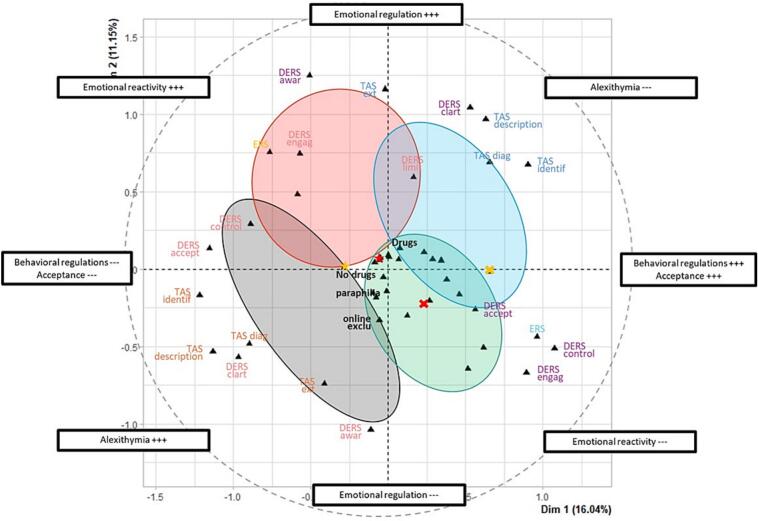
Multiple correspondence analysis factor map of the emotional-level profiles. *Note.* MCA = Multiple Correspondence Analysis; DERS = Difficulties in Emotion Regulation Scale; ERS = Emotion Reactivity Scale; TAS = Toronto Alexithymia Scale; DERS awar = awareness; DERS clart = clarity; DERS accept = nonacceptance/acceptance; DERS control = impulse control difficulties; DERS engag = difficulties engaging in goal-directed behavior; DERS limit = limited access to emotion regulation strategies; TAS identif = difficulty identifying feelings; TAS description = difficulty describing feelings; TAS ext = externally oriented thinking; no drugs/drugs = sexual behavior not associated vs. associated with psychoactive substance use; online exclu = exclusive cybersexual behavior; paraphilia = paraphilic practices.

At the emotional level, a four-group descriptive solution was observed (Fig. 2; Supplementary Information 2). Two profiles were marked by more pronounced emotional difficulties.

The first profile, termed *emotion-processing-limited* (n = 24; 31.6%), was characterized by marked alexithymia and difficulties in emotional awareness, clarity, and acceptance. Specifically, 95.8% of participants in this group fell in the diagnostic range for TAS total score, 70.8% were in the highest quartile for difficulty identifying feelings, and 91.7% were in the highest quartile for difficulty describing feelings. This group also showed elevated difficulties in emotional clarity and acceptance. These findings are consistent with a profile marked by limited emotional identification and symbolic processing.

The second profile, termed *emotionally reactive* (n = 13; 17.1%), was distinguished by heightened emotional reactivity and difficulties controlling behavior under emotional load. In this group, 76.9% of participants were in the highest quartile on the ERS, 61.5% in the highest quartile for DERS-control, and 92.3% in the highest quartile for difficulties engaging in goal-directed behavior. This profile therefore appears more strongly characterized by emotional intensity and affect-driven loss of control than by deficits in emotional identification per se.

Two additional profiles reflected more moderate or relatively preserved emotional functioning: the globally moderate regulation profile (n = 19; 25.0%), characterized by intermediate scores across most DERS and TAS dimensions and the absence of either marked alexithymic features or pronounced emotional reactivity; and the emotionally regulated profile (n = 20; 26.3%), characterized by relatively preserved emotional functioning, with the lowest levels of alexithymia, no participants in the highest ERS quartile, and the highest proportion of SAST-negative participants (55.0%). Emotional profiles were not associated with specific sexual practices.

#### 3.3.2 Cognitive profiles

Cognitive and metacognitive variables did not differ significantly according to practice type or substance use, except that exclusively cybersexual participants showed fewer relief-oriented distortions (p < 0.05) (Table 6).

**Table 6:** Univariate Associations for Cognitive-Level Variables.

Variables (rank)		Cyber			Paraphilia		PaS associated	
		None	Mixt	Exclusive	No	Yes	No	Yes
	TOTAL	37 (48.7)	28 (36.8)	11 (14.5)	49 (64.5)	27 (35.5)	54 (71.1)	22 (28.9)
**ASP-sex anticipatory(0 > 40)**	mean ± sd	16.6 ± 8.8	13.5 ± 7.1	12 ± 7.8	15.6 ± 8	13.3 ± 8.4	15.6 ± 8.6	12.8 ± 6.8
	median [Q1; Q3]	16 [10; 24]	13 [7.5; 17]	13 [6; 16]	15 [10; 20]	13 [6; 17]	14.5 [8; 23]	12 [8; 17]
**ASP-sex relief(0 > 40)**	mean ± sd	22.8 ± 8.5 *	19.6 ± 9 *	15.5 ± 8.5 *	22.3 ± 8.1	17.4 ± 9.6	20.4 ± 9.6	21 ± 7.1
	median [Q1; Q3]	26 [13; 28] *	19 [14.5; 19] *	16 [8; 24] *	24 [15; 28]	17 [8–26]	21 [13; 28]	23 [15; 26]
**ASP-sex permissive(0 > 40)**	mean ± sd	7.3 ± 2.8	8.9 ± 2.6	7.8 ± 2.4	8.1 ± 2.9	7.8 ± 2.5	7.8 ± 2.5	8.3 ± 3.2
	median [Q1; Q3]	7 [5; 8]	8 [7; 10.5]	7 [6; 9]	8 [6; 10]	7 [6; 10]	8 [6; 9]	8 [6; 10]
**BCIS self-reflexivity(0 > 27)**	mean ± sd	8.2 ± 3.1	7.8 ± 2.1	7 ± 2.3	7.7 ± 2.7	8.1 ± 2.6	7.8 ± 2.6	8 ± 3
	median [Q1; Q3]	8 [6; 10]	8 [6; 9]	7 [4; 8]	8 [6; 9]	8 [6; 10]	8 [6; 9]	8 [5; 9]
**BCIS self-certain(0 > 18**	mean ± sd	11.1 ± 3	11 ± 2.8	9.5 ± 3.7	10.8 ± 2.9	10.9 ± 3.4	10.5 ± 3	10.6 ± 3.1
	median [Q1; Q3]	11 [9; 13]	11 [9; 13.5]	9 [7; 13]	10 [9; 13]	11 [8; 14]	12 [9; 14]	11 [8; 13]
**BCIS TOTAL(−18 > 27)**	mean ± sd	11.2 ± 2.7	12.1 ± 2.8	10.8 ± 3.2	11.2 ± 2.7	11.8 ± 2.9	11.3 ± 3	11.9 ± 2.1
	median [Q1; Q3]	11 [9; 13]	13 [9.5; 14.5]	11 [9; 13]	12 [9; 13]	13 [10; 14]	11 [9; 14]	12 [11; 13]

*Note.* ASP-sex = adapted Anticipatory–Relief–Permissive cognitive distortions scale for sexual behavior; anticipatory = anticipatory cognitions; relief = relief-oriented cognitions; permissive = permissive cognitions; BCIS = Beck Cognitive Insight Scale; self-reflexivity = self-reflectiveness; self-certain = self-certainty; total = composite cognitive insight index; PaS = psychoactive substance-associated sexual behavior; Cyber none = no cybersexual behavior; Cyber mixt = mixed cybersexual and non-cybersexual behavior; Cyber exclusive = exclusive cybersexual behavior. *p < 0.05; **p < 0.01.

**Fig. 3:**
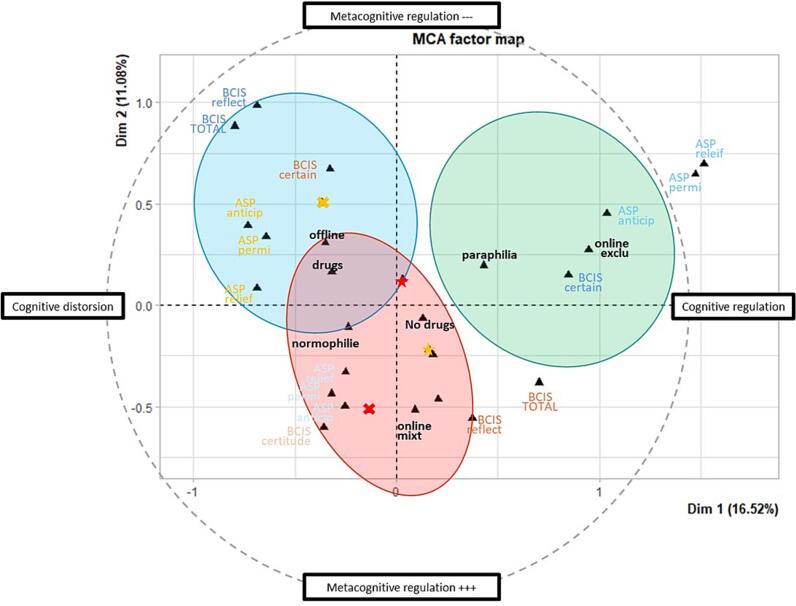
Multiple correspondence analysis factor map of the cognitive-level profiles. *Note.* MCA = Multiple Correspondence Analysis; ASP = Anticipatory–Relief–Permissive cognitive distortions scale (*Anticipatoire–Soulageante–Permissive* in French); BCIS = Beck Cognitive Insight Scale; BCIS reflect = self-reflectiveness; BCIS certain = self-certainty; BCIS total = composite cognitive insight index; no drugs/drugs = sexual behavior not associated vs. associated with psychoactive substance use; offline = non-cybersexual behavior; online exclu = exclusive cybersexual behavior; online mixt = mixed cybersexual and non-cybersexual behavior; normophilia/paraphilia = normophilic vs. paraphilic practices.

At the cognitive level, a three-group descriptive solution was retained (Fig. 3; Supplementary Information 3).

The first profile, termed *high-insight* (n = 18; 23.7%), was characterized by the lowest levels of cognitive distortions and comparatively preserved insight. Most participants in this group fell in the lowest quartile for anticipatory (83.3%), relief-oriented (83.3%), and permissive cognitions (88.9%). At the same time, BCIS indices suggested relatively preserved cognitive insight, and this profile was the one most frequently associated with paraphilic practices (55.6%). This configuration is consistent with greater metacognitive distance from maladaptive cognitions related to sexual behavior.

The second profile, termed *low-insight* (n = 22; 28.9%), showed the lowest cognitive insight and the most maladaptive metacognitive pattern. All participants in this group fell in the lowest category of BCIS total score, 72.7% were in the lowest quartile for self-reflectiveness, and 59.1% were in the highest quartile for self-certainty. This group also showed relatively elevated cognitive distortions, particularly anticipatory and permissive cognitions. In addition, it included the highest proportion of SAST-negative participants (59.1%), suggesting a profile marked less by acknowledged symptom severity than by reduced cognitive distance from maladaptive beliefs.

An intermediate *average regulation* profile (n = 36; 47.4%) displayed scores close to the sample mean across most cognitive variables, with the majority of participants falling in the middle quartiles for anticipatory, relief-oriented, and permissive cognitions, as well as for BCIS indices. Overall, cognitive profiles were only weakly associated with behavioral modalities.

Bootstrap-based stability analysis indicated consistent and overall satisfactory robustness of the clustering solutions across the three analyses. Jaccard similarity coefficients ranged from 0.64 to 0.81 for Fig. 1, from 0.80 to 0.92 for Fig. 2, and from 0.94 to 0.95 for Fig. 3, corresponding to moderate, good to excellent, and excellent stability, respectively. All values exceeded commonly accepted thresholds for non-random cluster structure, with particularly high reproducibility observed in the third analysis. These findings were further supported by sensitivity analyses, which yielded consistent clustering patterns across alternative specifications.

## 4 Discussion

The present exploratory study examined whether distinct descriptive configurations could be observed at motivational, emotional, and cognitive levels among adults reporting DSB. In this small heterogeneous sample, descriptive groupings were observed within each of these domains, suggesting that subjective loss of control over sexual behavior may be associated with multiple psychological configurations rather than a single uniform pattern. These findings should be interpreted cautiously. They do not validate a formal typological model, nor do they establish stable clinical subtypes. Rather, they suggest that a multilevel descriptive perspective may be useful for documenting heterogeneity within DSB (Grubbs et al., 2020).

### 4.1 Behavioral heterogeneity and functional meaning

A first noteworthy result is that the behavioral modalities considered in this study were only weakly associated with the psychological variables examined. In this sample, paraphilic versus normophilic practices, cybersexual activity, and sexual behavior associated with psychoactive substance use provided relatively limited information about the broader motivational, emotional, and cognitive configurations observed. This finding is clinically and conceptually relevant because it suggests that overt behavioral content alone may not adequately capture the processes associated with DSB. If replicated, such a pattern would support the value of process-oriented formulations that attend less exclusively to the form of sexual behavior and more closely to the functions with which it is associated.

At the motivational or dynamic level, the three observed profiles may be understood as reflecting different possible functional orientations within DSB. One profile was characterized primarily by sensation seeking and positive urgency, and may be consistent with a greater role of novelty, excitement, and immediate reward in the reported behaviors. A second profile combined anxiety, depressive symptoms, and negative urgency, and may reflect a stronger association between sexual behavior and distress-related coping processes. A third profile was characterized by relatively preserved psychological functioning together with more marked cognitive distortions, and may suggest the relevance of more habitual or cognitively maintained patterns. These interpretations remain tentative, but they suggest that a single motivational account is unlikely to capture the full heterogeneity of DSB.

The resemblance between these motivational configurations and pathways-oriented perspectives described in the broader addiction literature may be heuristically informative (Blaszczynski and Nower, 2002, Golder et al., 2023, Nower et al., 2022). However, the present findings should not be interpreted as validating a pathways model of DSB, nor as demonstrating that motivational processes in DSB are organized according to the same structure as in gambling disorder. The value of this comparison is primarily descriptive: it highlights the possibility that similar clinical outcomes may be associated with partially distinct motivational configurations. In that sense, the present results are more useful for generating hypotheses than for adjudicating between competing theoretical models.

### 4.2 Emotional and cognitive differentiation

At the emotional level, the findings also pointed to non-uniform forms of dysregulation. One profile was marked more strongly by alexithymia and difficulties in identifying, clarifying, and accepting emotional states (Gori and Topino, 2024, Preece et al., 2023), whereas another was marked more strongly by heightened emotional reactivity and difficulty maintaining behavioral control in emotionally salient contexts. These configurations suggest that emotional difficulties in DSB may not be reducible to a single dimension (Lew-Starowicz et al., 2020). For some individuals, the central difficulty may lie more in limited emotional awareness and symbolic elaboration; for others, it may lie more in emotional intensity and affect-driven loss of control. The presence of more moderate or relatively preserved emotional profiles further cautions against assuming that emotional dysregulation is uniformly central across all individuals reporting DSB.

At the cognitive level, the observed profiles suggest that maladaptive cognitions and cognitive insight may also vary meaningfully across individuals reporting DSB (Efrati et al., 2021). The low-insight profile may reflect greater difficulty in questioning or regulating maladaptive beliefs related to sexual behavior (Penzenstadler et al., 2020), whereas the high-insight profile may reflect relatively greater metacognitive distance from such cognitions. The fact that the high-insight profile was more frequently observed among participants reporting paraphilic practices may invite further reflection on the possible role of self-monitoring, social norm awareness, or stigma in shaping cognitive regulation (Jahnke et al., 2015). This interpretation should remain cautious, but it suggests that cognitive functioning may add a distinct descriptive layer to the understanding of heterogeneity in DSB.

### 4.3 Clinical implications of a multilevel descriptive approach

Taken together, the present findings illustrate the heuristic value of distinguishing motivational, emotional, and cognitive domains when exploring DSB. In this exploratory study, the multilevel descriptive strategy made it possible to preserve interpretive clarity across a heterogeneous sample and to capture configurations that a single global model might have obscured. These domains are not independent, and the present work does not claim to establish their boundaries definitively. What it does suggest is that heterogeneity in DSB may be clinically and theoretically meaningful at each of these levels — and that mapping this heterogeneity, however tentatively, may constitute a useful starting point for more differentiated clinical and empirical work.

These considerations point, in turn, to the clinical relevance of the present framework, however preliminary. The clinical implications of these findings should remain modest and provisional. The observed profiles should not be understood as stable clinical subtypes or as a basis for prescriptive treatment algorithms. Nevertheless, they may carry some practical value at the level of case formulation. The results suggest that individuals reporting DSB may differ in the relative prominence of motivational, emotional, and cognitive difficulties — and that attending to this heterogeneity may help clinicians identify which functional dimensions warrant priority at a given stage of care. Rather than constituting a prescriptive protocol, this perspective supports a process-informed and individualized approach to case formulation (Briken et al., 2024, Griffin et al., 2021). To illustrate how such a multilevel rationale might translate into clinical reasoning, we developed a preliminary heuristic framework mapping the functional profiles identified at each level onto corresponding axes of care (Supplementary Information 4). This framework is explicitly not a treatment algorithm, nor does it presuppose a specific nosographic position. It is offered as a clinical reasoning aid – a way of structuring the question of which therapeutic dimensions may deserve attention first, depending on the configuration observed in a given patient.

### 4.4 Limitations and future directions

Several limitations should be acknowledged. First, the study relied on a small and heterogeneous sample recruited through both clinical and non-clinical channels. The resulting profiles should therefore be interpreted as exploratory and sample-dependent rather than as robust subtypes. However, although the sample size was relatively modest, the bootstrap stability analysis suggests that the identified clustering structures are not purely sample-specific. Stability varied across analyses, with some clusters showing moderate robustness and others demonstrating excellent reproducibility. These findings support the internal coherence of the typologies, while still warranting cautious interpretation and replication in larger samples. Second, all measures were self-reported, which may have introduced reporting biases, especially in a field where shame, minimization, and moral conflict may affect disclosure. Third, the clustering procedures were conducted separately at motivational, emotional, and cognitive levels using the same participants. Although this choice was consistent with the exploratory and clinically grounded aims of the study, it does not establish the statistical independence of these domains or validate a multilevel typological structure. Future studies might also consider integrative analytical approaches — such as latent profile analysis or network models — that would allow the simultaneous modelling of motivational, emotional, and cognitive variables while preserving their distinct contributions. Fourth, the profile labels were heuristic and should not be reified as stable clinical entities. Fifth, the cross-sectional design precludes causal or developmental interpretations. A further point of caution concerns the use of instruments historically developed within the sexual addiction literature, particularly the SAST. In the present study, such measures were retained for pragmatic reasons in order to document clinically relevant manifestations of self-reported DSB. Their use should not be interpreted as an endorsement of an addiction-based ontology. Similarly, craving was treated here as a clinically relevant experiential dimension of urge and loss of control, rather than as evidence for a specifically addictive model of DSB. Finally, the adapted ASP-Sex scale has not undergone full psychometric validation, and findings involving this measure should therefore be considered preliminary.

Accordingly, the present results should be understood as descriptive and hypothesis-generating. They document possible forms of heterogeneity within DSB, but they do not establish a definitive typology or clinically validated classification. Replication in larger, better-characterized samples will be necessary before stronger conceptual or clinical claims can be made.

## 5 Conclusion

In this exploratory study, DSB appeared less as a single homogeneous clinical phenomenon than as a set of partially distinct psychological configurations observable across motivational, emotional, and cognitive domains. Although the present findings do not establish a validated typology, they highlight the potential value of a multilevel descriptive approach for documenting heterogeneity in individuals reporting loss of control over sexual behavior.

One of the main contributions of this work lies in showing that overt behavioral modalities – whether paraphilic or normophilic, cybersexual or not, associated with psychoactive substance use or not – may provide only limited information about the broader psychological processes involved. By contrast, examining DSB through complementary domains of functioning makes it possible to generate more differentiated and clinically meaningful hypotheses regarding the role of reward seeking, distress regulation, emotional processing, emotional reactivity, maladaptive cognitions, and cognitive insight.

From this perspective, the study offers a clinically useful shift from behavior-centered description toward process-informed case formulation. Its value lies not in proposing a definitive explanatory model, but in suggesting that heterogeneity in DSB may be more fruitfully approached through the identification of distinct functional configurations that each warrant careful assessment. In this sense, the present findings may help support more individualized clinical thinking and provide a heuristic basis for future research on mechanisms, trajectories, and intervention needs in this field.

Further studies in larger and better-characterized samples, using strengthened statistical procedures and ideally longitudinal designs, will be needed to determine the reproducibility and clinical utility of the descriptive profiles identified here. Nonetheless, the present work contributes to ongoing efforts to better understand the complexity of DSB by offering a coherent exploratory framework for examining its psychological heterogeneity.

## 6 Authors’ contribution

CM: made substantial contributions to the conception of the work; made substantial contributions to the acquisition, analysis, and interpretation of data; drafted the work for important intellectual content; agree to be accountable for all aspects of the work in ensuring that questions related to the accuracy or integrity of any part of the work are appropriately investigated and resolved.

JC: revised it critically for important intellectual content; approved the version to be published; agree to be accountable for all aspects of the work in ensuring that questions related to the accuracy or integrity of any part of the work are appropriately investigated and resolved.

BP: made substantial contributions to the analysis, and interpretation of data; approved the version to be published; agree to be accountable for all aspects of the work in ensuring that questions related to the accuracy or integrity of any part of the work are appropriately investigated and resolved.

VM: made substantial contributions to the conception of the work; made substantial contributions to the analysis, and interpretation of data; revised it critically for important intellectual content; approved the version to be published; agree to be accountable for all aspects of the work in ensuring that questions related to the accuracy or integrity of any part of the work are appropriately investigated and resolved.

SB: made substantial contributions to the conception of the work; made substantial contributions to the analysis, and interpretation of data; revised it critically for important intellectual content; approved the version to be published; agree to be accountable for all aspects of the work in ensuring that questions related to the accuracy or integrity of any part of the work are appropriately investigated and resolved.

## 7 Founding source

No financial support was received for this study.

## 8 Consent to participate

Informed consent was obtained from all individual participants included in the study.

## Credit authorship contribution statement

**Cécile Miele:** Writing – original draft, Project administration, Methodology, Investigation, Data curation, Conceptualization. **Julien Cabé:** Validation, Supervision, Investigation, Data curation. **Bruno Pereira:** Validation, Software, Methodology, Formal analysis, Conceptualization. **Valérie Moulin:** Visualization, Validation, Supervision, Resources, Methodology, Conceptualization. **Servane Barrault:** Visualization, Validation, Supervision, Resources, Methodology, Conceptualization.

## Declaration of competing interest

The authors declare the following financial interests/personal relationships which may be considered as potential competing interests: Cecile Miele reports was provided by University Hospital Centre Clermont-Ferrand. Cecile Miele reports a relationship with University Hospital Centre Clermont-Ferrand that includes: employment. If there are other authors, they declare that they have no known competing financial interests or personal relationships that could have appeared to influence the work reported in this paper.

## Data Availability

Data will be made available on request.
