# Supplementary figures and images for: Motivational, emotional, and cognitive profiles of dysregulated sexual behavior: a multilevel exploratory study
Source: Addict Behav Rep. 2026 Apr 12;23:100696. doi: 10.1016/j.abrep.2026.100696 (PMC13101632; doi:10.1016/j.abrep.2026.100696)

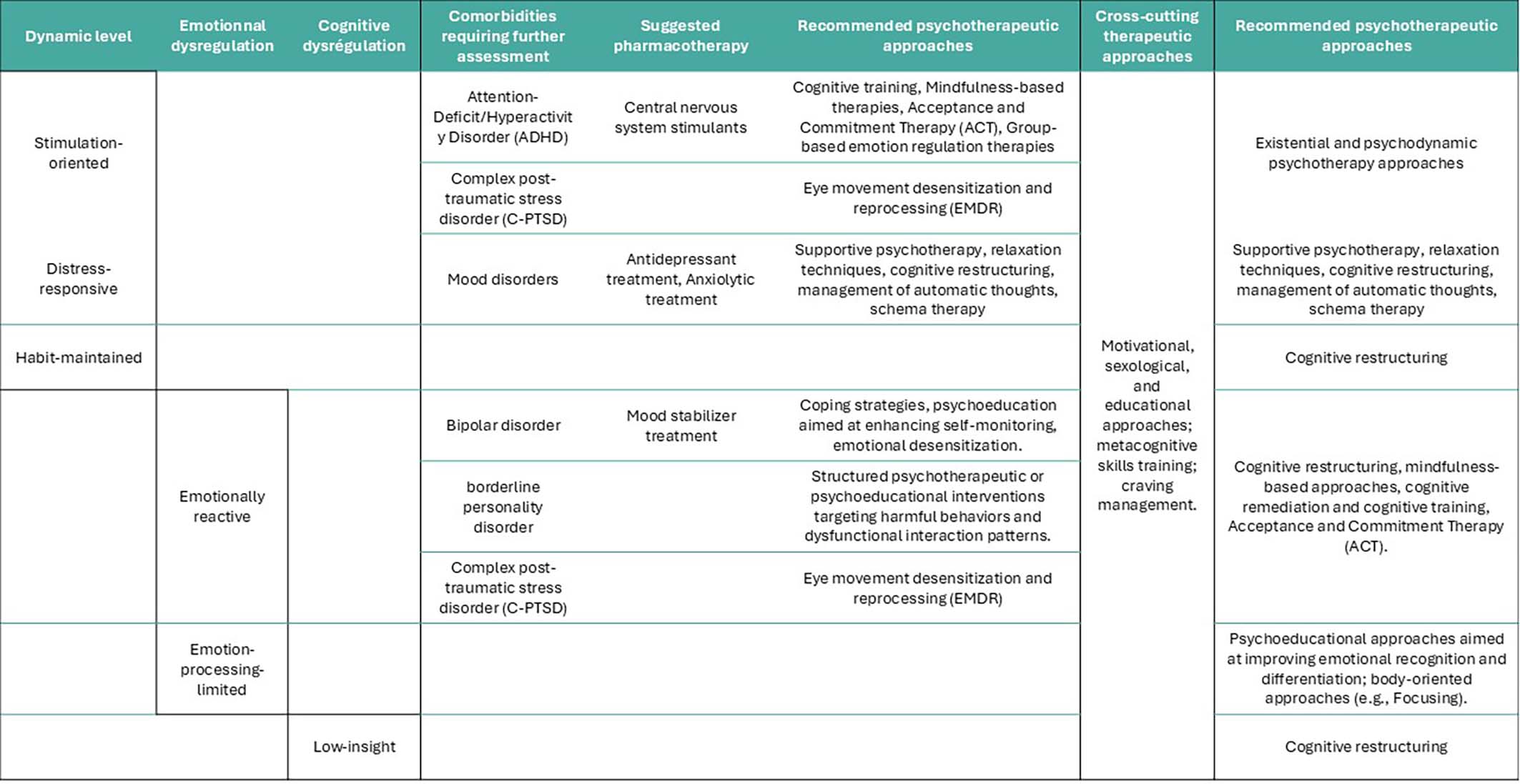

Supplement: Supplementary Fig. 1 [file mmc4.jpg]
