# Supplementary material for: Motivational, emotional, and cognitive profiles of dysregulated sexual behavior: a multilevel exploratory study
Source: Addict Behav Rep. 2026 Apr 12;23:100696. doi: 10.1016/j.abrep.2026.100696 (PMC13101632; doi:10.1016/j.abrep.2026.100696)
Supplement: Supplementary Data 1 [file mmc1.docx]

SuppInfo 1. Variable contribution to each cluster at the dynamic level

| Variables | Category | TOTAL  n (%) | Cluster 1 | Cluster 2 | Cluster 3 | p-value |
| --- | --- | --- | --- | --- | --- | --- |
|  |  |  | n (%) | n (%) | n (%) |  |
|  |  |  | 44 (57.9) | 13 (17.1) | 19 (25) |  |
| PaS | no | 54 (71.1) | 33 (75) | 8 (61.5) | 13 (68.4) | 0.616 |
|  | yes | 22 (29) | 11 (25) | 5 (38.5) | 6 (31.6) |  |
| Cybersex | no | 37 (48.7) | 22 (50) | 5 (38.5) | 10 (52.6) | 0.716 |
|  | mixt | 28 (36.8) | 15 (34.1) | 7 (53.9) | 6 (31.6) |  |
|  | exclusive | 11 (14.5) | 7 (15.9) | 1 (7.7) | 3 (15.8 |  |
| Paraphilia | no | 49 (64.5) | 30 (68.2) | 7 (53.9) | 12 (63.2) | 0.631 |
|  | yes | 27 (35.5) | 14 (31.8) | 6 (46.2) | 7 (36.8) |  |
| HAD – Depression | none | 41 (54) | 38 (86.4) | 0 (0) | 3 (15.8) | 0.001 |
|  | doubt | 17 (22.4) | 5 (11.4) | 12 (92.3) | 0 (0) |  |
|  | diagnostic | 18 (23.7) | 1 (2.3) | 1 (7.7) | 16 (84.2) |  |
| HAD – Anxiety | none | 23 (30.3) | 21 (47.7) | 1 (7.7) | 1 (2.3) | 0.001 |
|  | doubt | 20 (26.3) | 15 (34.1) | 5 (38.5) | 0 (0) |  |
|  | diagnostic | 33 (43.4) | 8 (18.2) | 7 (53.9) | 18 (94.7) |  |
| HAD – Anxiety+Depression | none | 48 (63.2) | 44 (100) | 1 (7.7) | 3 (15.8) | 0.001 |
|  | 2 subsacles > doubt | 12 (15.8) | 0 (0) | 12 (92.3) | 0 (0) |  |
|  | 2 subscales > diag | 16 (21.1) | 0 (0) | 0 (0) | 16 (84.2) |  |
| UPPS-P – Nega Urg | Q1 | 20 (26.3) | 17 (38.6) | 2 (15.4) | 1 (5.3) | 0.006 |
|  | Q2 + Q3 | 35 (46.1) | 21 (47.7) | 6 (46.2) | 8 (42.1) |  |
|  | Q4 | 21 (27.6) | 6 (13.6) | 5 (38.5) | 10 (52.6) |  |
| UPPS-P – Persev | Q1 | 25 (32.9) | 15 (34.1) | 4 (30.8) | 6 (31.6) | 0.335 |
|  | Q2 + Q3 | 31 (40.8) | 21 (47.7) | 5 (38.5) | 5 (26.3) |  |
|  | Q4 | 20 (26.3) | 8 (18.2) | 4 (30.8) | 8 (42.1) |  |
| UPPS-P – Prem | Q1 | 24 (31.6) | 18 (40.9) | 4 (30.8) | 2 (10.5) | 0.013 |
|  | Q2 + Q3 | 23 (30.3) | 16 (36.4) | 3 (23.1) | 4 (21.1) |  |
|  | Q4 | 29 (38.2) | 10 (22.7) | 6 (46.2) | 13 (68.4) |  |
| UPPS-P – Sensa | Q1 | 5 (6.6) | 2 (4.6) | 0 (0) | 3 (15.8) | 0.406 |
|  | Q2 + Q3 | 24 (31.6) | 15 (34.1) | 4 (30.8) | 5 (26.3) |  |
|  | Q4 | 47 (61.8) | 27 (61.4) | 9 (69.23) | 11 (57.9) |  |
| UPPS-P – Posi Urg | Q1 | 22 (29) | 15 (34.1) | 4 (30.8) | 3 (15.8) | 0.258 |
|  | Q2 + Q3 | 20 (26.3) | 17 (38.6) | 1 (7.7) | 7 (36.8) |  |
|  | Q4 | 34 (44.7) | 17 (38.6) | 8 (61.5) | 9 (47.4) |  |
| ASP-sex – Anticip | Q1 | 22 (29) | 7 (15.9) | 4 (30.8) | 11 (57.9) | 0.012 |
|  | Q2 + Q3 | 35 (46.1) | 23 (52.3) | 5 (38.5) | 7 (36.8) |  |
|  | Q4 | 19 (25) | 14 (31.8) | 4 (30.8) | 1 (5.3) |  |
| ASP-sex – Releif | Q1 | 16 (21.1) | 5 (11.4) | 2 (15.4) | 9 (47.4) | 0.029 |
|  | Q2 + Q3 | 39 (51.3) | 26 (59.1) | 7 (53.9) | 6 (31.6) |  |
|  | Q4 | 21 (27.6) | 13 (29.6) | 4 (30.8) | 4 (21.1) |  |
| SAST | no | 23 (30.3) | 20 (45.5) | 1 (7.7) | 2 (10.5) | 0.003 |
|  | yes | 53 (69.7) | 24 (54.6) | 12 (92.3) | 17 (89.5) |  |
| Craving | no | 15 (19.7) | 13 (29.6) | 1 (7.7) | 1 (5.3) | 0.041 |
|  | yes | 61 (80.3) | 31 (70.5) | 12 (92.3) | 18 (94.7) |  |
