# Supplementary material for: Motivational, emotional, and cognitive profiles of dysregulated sexual behavior: a multilevel exploratory study
Source: Addict Behav Rep. 2026 Apr 12;23:100696. doi: 10.1016/j.abrep.2026.100696 (PMC13101632; doi:10.1016/j.abrep.2026.100696)
Supplement: Supplementary Data 2 [file mmc2.docx]

SuppInfo 2. Variable contribution to each cluster at the emotional level

| Variable | Category | TOTAL  n (%) | Cluster 1 | Cluster 2 | Cluster 3 | Cluster 4 | p-value |
| --- | --- | --- | --- | --- | --- | --- | --- |
|  |  |  | n (%) | n (%) | n (%) | n (%) |  |
|  |  |  | 24 (31.6) | 13 (17.1) | 19 (25) | 20 (26.3) |  |
| PaS | no | 54 (71.1) | 17 (70.8) | 11 (84.6) | 14 (73.7) | 12 (60) | 0.491 |
|  | yes | 22 (29) | 7 (29.2) | 2 (15.4) | 5 (26.3) | 8 (40) |  |
| Cybersex | no | 37 (48.7) | 14 (58.3) | 5 (38.5) | 5 (26.3) | 13 (65) | 0.207 |
|  | mixt | 28 (36.8) | 7 (29.2) | 6 (46.2) | 9 (47.4) | 6 (30) |  |
|  | exclusive | 11 (14.5) | 3 (12.5) | 2 (15.4) | 5 (26.3) | 1 (5) |  |
| Paraphilia | no | 49 (64.5) | 14 (58.3) | 9 (69.2) | 12 (63.2) | 14 (70) | 0.848 |
|  | yes | 27 (35.5) | 10 (41.7) | 4 (30.8) | 7 (36.8) | 6 (30) |  |
| DERS – Clarty | Q1 | 15 (19.7) | 0 (0) | 4 (30.8) | 0 (0) | 11 (55) | 0.000 |
|  | Q2 + Q3 | 38 (50) | 4 (16.7) | 9 (69.2) | 17 (89.5) | 8 (40) |  |
|  | Q4 | 23 (30.3) | 20 (83.3) | 0 (0) | 2 (10.5) | 1 (5) |  |
| DERS – Awarness | Q1 | 14 (18.4) | 3 (12.5) | 9 (69.2) | 0 (0) | 2 (10) | 0.000 |
|  | Q2 + Q3 | 41 (54) | 9 (37.5) | 4 (30.8) | 11 (57.9) | 17 (85) |  |
|  | Q4 | 21 (27.6) | 12 (50) | 0 (0) | 8 (42.1) | 1 (5) |  |
| DERS – Acceptance | Q1 | 18 (23.7) | 5 (20.8) | 1 (7.7) | 4 (21.1) | 8 (40) | 0.000 |
|  | Q2 + Q3 | 38 (50) | 5 (20.8) | 7 (53.9) | 15 (79) | 11 (55) |  |
|  | Q4 | 20 (26.3) | 14 (58.3) | 5 (38.5) | 0 (0) | 1 (5) |  |
| DERS – Control | Q1 | 19 (25) | 2 (8.3) | 0 (0) | 8 (42.1) | 9 (45) | 0.000 |
|  | Q2 + Q3 | 34 (44.7) | 10 (41.7) | 5 (38.5) | 10 (52.6) | 9 (45) |  |
|  | Q4 | 23 (30.3) | 12 (50) | 8 (61.5) | 1 (5.3) | 2 (10) |  |
| DERS – Engag | Q1 | 19 (25) | 2 (8.3) | 0 (0) | 10 (52.6) | 7 (35) | 0.000 |
|  | Q2 + Q3 | 33 (43.4) | 14 (58.3) | 1 (7.7) | 7 (36.8) | 11 (55) |  |
|  | Q4 | 24 (31.6) | 8 (33.3) | 12 (92.3) | 2 (10.5) | 2 (10) |  |
| DERS – Limit | Q1 | 19 (25) | 5 (20.8) | 5 (38.5) | 4 (21.1) | 5 (25) | 0.134 |
|  | Q2 + Q3 | 38 (50) | 14 (58.3) | 5 (38.5) | 13 (68.4) | 6 (30) |  |
|  | Q4 | 19 (25) | 5 (20.8) | 3 (23.1) | 2 (10.5) | 9 (45) |  |
| ERS | Q1 | 19 (25) | 3 (12.5) | 0 (0) | 7 (36.8) | 9 (45) | 0.000 |
|  | Q2 + Q3 | 37 (48.7) | 14 (58.3) | 3 (23.1) | 9 (47.4) | 11 (55) |  |
|  | Q4 | 39 (51.3) | 7 (29.2) | 10 (76.9) | 3 (15.8) | 0 (0) |  |
| TAS – Identif | Q1 | 17 (22.4) | 0 (0) | 2 (15.4) | 2 (10.5) | 13 (65) | 0.000 |
|  | Q2 + Q3 | 38 (50) | 7 (29.2) | 8 (61.5) | 16 (84.2) | 7 (35) |  |
|  | Q4 | 21 (27.6) | 17 (70.8) | 3 (23.1) | 1 (5.3) | 0 (0) |  |
| TAS – Descrip | Q1 | 19 (25) | 0 (0) | 5 (38.5) | 0 (0) | 14 (70) | 0.000 |
|  | Q2 + Q3 | 33 (43.4) | 2 (8.3) | 6 (46.2) | 19 (100) | 6 (30) |  |
|  | Q4 | 24 (31.6) | 22 (91.7) | 2 (15.4) | 0 (0) | 0 (0) |  |
| TAS – Ext | Q1 | 14 (18.4) | 2 (8.3) | 6 (46.2) | 0 (0) | 6 (30) | 0.000 |
|  | Q2 + Q3 | 37 (48.7) | 7 (29.2) | 6 (46.2) | 13 (68.4 | 11 (55) |  |
|  | Q4 | 25 (32.9) | 15 (62.5) | 1 (7.7) | 6 (31.6) | 3 (15) |  |
| TAS - Total | none | 33 (43.42) | 0 (0) | 9 (69.2) | 5 (26.3) | 19 (95) | 0.000 |
|  | doubt | 12 (15.8) | 1 (4.2) | 0 (0) | 11 (57.9) | 0 (0) |  |
|  | diagnostic | 31 (40.8) | 23 (95.8) | 4 (30.8) | 3 (15.8) | 1 (5) |  |
| SAST | no | 23 (30.3) | 2 (8.3) | 0 (0) | 10 (52.6) | 11 (55) | 0.000 |
|  | yes | 53 (69.7) | 22 (91.7) | 13 (100) | 9 (47.4) | 9 (45) |  |
| Craving | no | 15 (19.7) | 4 (16.7) | 0 (0) | 4 (21.1) | 7 (35) | 0.098 |
|  | yes | 61 (80.3) | 20 (83.3) | 13 (100) | 15 (79) | 13 (65) |  |
