# Supplementary material for: Motivational, emotional, and cognitive profiles of dysregulated sexual behavior: a multilevel exploratory study
Source: Addict Behav Rep. 2026 Apr 12;23:100696. doi: 10.1016/j.abrep.2026.100696 (PMC13101632; doi:10.1016/j.abrep.2026.100696)
Supplement: Supplementary Data 3 [file mmc3.docx]

SuppInfo 3. Variable contribution to each cluster at the cognitive level

| Variable | Category | TOTAL  n (%) | Cluster 1 | Cluster 2 | Cluster 3 | p-value |
| --- | --- | --- | --- | --- | --- | --- |
|  |  |  | n (%) | n (%) | n (%) |  |
|  |  |  | 36 (47.4) | 18 (23.7) | 22 (28.9) |  |
| PaS | no | 54 (71.1) | 26 (72.2) | 14 (77.8) | 14 (63.6) | 0.604 |
|  | yes | 22 (29) | 10 (27.8) | 4 (22.2) | 8 (36.4) |  |
| Cybersex | no | 37 (48.7) | 17 (47.2) | 7 (38.9) | 13 (59.1) | 0.377 |
|  | mixt | 28 (36.8) | 15 (41.7) | 6 (33.3) | 7 (31.8) |  |
|  | exclusive | 11 (14.5) | 4 (11.1) | 5 (27.8) | 2 (9.1) |  |
| Paraphilia | no | 49 (64.5) | 28 (77.8) | 8 (44.4) | 13 (59.1) | 0.045 |
|  | yes | 27 (35.5) | 8 (22.2) | 10 (55.6) | 9 (40.9) |  |
| ASP-sex – Anticip | Q1 | 22 (29) | 4 (11.1) | 15 (83.3) | 3 (13.6) | 0.000 |
|  | Q2 + Q3 | 35 (46.1) | 22 (61.1) | 3 (16.7) | 10 (45.5) |  |
|  | Q4 | 19 (25) | 10 (27.8) | 0 (0) | 9 (40.9) |  |
| ASP-sex – Releif | Q1 | 16 (21.1) | 1 (2.8) | 15 (83.3) | 0 (0) | 0.000 |
|  | Q2 + Q3 | 39 (51.3) | 21 (58.3) | 3 (16.7) | 15 (68.2) |  |
|  | Q4 | 21 (27.6) | 14 (38.9) | 0 (0) | 7 (31.8) |  |
| ASP-sex – Permi | Q1 | 17 (22.4) | 0 (0) | 16 (88.9) | 1 (4.6) | 0.000 |
|  | Q2 + Q3 | 40 (52.6) | 26 (72.2) | 2 (11.1) | 12.54.6) |  |
|  | Q4 | 19 (25) | 10 (27.8) | 0 (0) | 9 (40.9) |  |
| BCIS – Self-reflex | Q1 | 20 (26.3) | 2 (5.6) | 2 (11.1) | 16 (72.7) | 0.000 |
|  | Q2 + Q3 | 37 (48.7) | 21 (58.3) | 11 (61.1) | 5 (22.7) |  |
|  | Q4 | 19 (25) | 13 (36.1) | 5 (27.8) | 1 (4.6) |  |
| BCIS – Self-certain | Q1 | 22 (29) | 10 (27.8) | 11 (61.1) | 1 (4.6) | 0.000 |
|  | Q2 + Q3 | 31 (40.8) | 21 (58.3) | 2 (11.1) | 8 (36.4) |  |
|  | Q4 | 23 (30.3) | 5 (13.9) | 5 (27.8) | 13 (59.1) |  |
| BCIS TOTAL | Q1 | 25 (32.9) | 1 (2.78) | 2 (11.1) | 22 (100) | 0.000 |
|  | Q2 + Q3 | 32 (42.1) | 24 (66.7) | 8 (44.4) | 0 (0) |  |
|  | Q4 | 19 (25) | 11 (30.6) | 8 (44.4) | 0 (0) |  |
| SAST | no | 23 (30.3) | 6 (16.7) | 4 (22.2) | 13 (59.1) | 0.002 |
|  | yes | 53 (69.7) | 30 (83.3) | 14 (77.8) | 9 (40.9) |  |
| Craving | no | 15 (19.7) | 8 (22.2) | 3 (16.7) | 4 (18.2) | 0.869 |
|  | yes | 61 (80.3) | 28 (77.8) | 15 (83.3) | 18 (81.8) |  |
